# Association of sarcopenia with phase angle and body mass index in kidney transplant recipients

**DOI:** 10.1038/s41598-019-57195-z

**Published:** 2020-01-14

**Authors:** Akihiro Kosoku, Junji Uchida, Shunji Nishide, Kazuya Kabei, Hisao Shimada, Tomoaki Iwai, Keiko Maeda, Yoshiko Hanayama, Takuma Ishihara, Toshihide Naganuma, Yoshiaki Takemoto, Tatsuya Nakatani

**Affiliations:** 1 Osaka City University Graduate School of Medicine, Department of Urology, Osaka, 545-8585 Japan; 2 Osaka City University Hospital, Department of Nursing, Osaka, 545-8586 Japan; 3 Osaka City University Hospital, Department of Nutrition, Osaka, 545-8586 Japan; 4 Innovative and Clinical Research Promotion Center, Gifu University Hospital, Gifu, 501-1194 Japan

**Keywords:** Nutrition, Continuous renal replacement therapy

## Abstract

Malnutrition is an important risk factor for the development of sarcopenia. Recently, phase angle (PhA) obtained from the bioelectrical impedance analysis is increasingly becoming known as a nutritional status marker and may be considered a good indicator to identify elderly patients at risk of sarcopenia. In this study, we investigated the prevalence of sarcopenia and the relationship between sarcopenia and PhA or body mass index (BMI) as nutritional factors, and evaluated the discrimination performance of these nutritional factors for sarcopenia in 210 kidney transplant recipients. The median age was 55 years and 11.1% had sarcopenia. This prevalence of sarcopenia was lower than previous reports in kidney transplant recipients, maybe because of the differences in sarcopenia definitions and population demographics such as age, sex, race, and comorbidities. Both PhA and BMI were negatively correlated with sarcopenia after adjusting for age, sex, dialysis vintage, time after transplant, presence of diabetes mellitus, hemoglobin, estimated glomerular filtration rate, and the other nutritional factor. The discrimination performance for PhA and BMI had enough power to detect sarcopenia. These results suggest that PhA and BMI can be used in clinical practice to predict sarcopenia in kidney transplant patients.

## Introduction

Kidney transplantation is the optimal renal replacement therapy for end-stage kidney disease patients, enabling greater longevity and better quality of life compared with dialysis therapy, even for the elderly^1,2^. Meanwhile, the number of kidney transplants for elderly end-stage kidney disease patients is increasing, with improved graft and patient survivals^3,4^, consequently contributing to the aging of kidney transplant recipients.

Sarcopenia is a geriatric syndrome characterized by an age-related decline in skeletal muscle mass plus low muscle strength and/or physical performance according to the Asian Working Group for Sarcopenia (AWGS)^5^. It is associated with adverse clinical outcomes, which include falls, disability, hospital admission, poorer quality of life, and mortality^6–10^. Primary sarcopenia is caused by aging, while secondary sarcopenia is caused by low activity, malnutrition, and disease (organ failure, inflammatory disease, malignancy, and endocrine disease)^11^. Although kidney transplant recipients can recover their renal function after transplantation, most of them still have chronic kidney disease (CKD) as well as a gradual decline in renal graft function due to chronic allograft nephropathy. CKD patients are associated with many clinical causes of sarcopenia such as low physical activity, decreased food intake due to anorexia caused by uremic toxins and inflammation, urine and/or dialysate nutrient losses, catabolic and anabolic hormone dysfunction, metabolic acidosis, and chronic inflammation^12^. Osteoporosis is also a risk factor for sarcopenia, because sarcopenia and osteoporosis share common biological pathways and risk factors^13^. Therefore, kidney transplant recipients may be high-risk patients for sarcopenia due to risk factors including CKD, aging, and glucocorticoid-induced osteoporosis.

Malnutrition is an important risk factor for the development of sarcopenia^11^. Several methods are used for the assessment of nutritional status such as body mass index (BMI), which are often used in clinical practice. Recently, phase angle (PhA) is increasingly becoming known as a nutritional status indicator. PhA is a parameter obtained from the bioelectrical impedance analysis (BIA) which has been used as a cell health marker, and is associated with cell membrane integrity, mortality, diet quality, nutritional status, muscle mass, and muscle function^14–16^. Previous reports demonstrated that PhA may be considered a good marker to identify elderly patient at risk of sarcopenia^16,17^. However, factors associated with sarcopenia in kidney transplant patients remain unknown. The aim of the present study is twofold: firstly, to investigate the prevalence of sarcopenia and the relationship between sarcopenia and PhA or BMI as nutritional factors, and secondly, to evaluate the discrimination performance of these nutritional factors for sarcopenia in kidney transplant recipients.

## Results

A total of 210 kidney transplant recipients were enrolled in this study^18^. The median age was 55 (interquartile range (IQR) 45–66) years, 122 (58%) were male, and 47 (22%) had diabetes mellitus. The median dialysis vintage was 19 (IQR 6–67) months, and the median time after transplant was 85 (IQR 43–135) months. The median BMI and PhA were 22 (IQR 20–25) kg/m^2^ and 4.8 (4.4–5.3°), respectively. Table 1 shows the demographics, characteristics, and clinical data for the participants, and comparisons between the sarcopenia group (n = 24, 11%) and non-sarcopenia group (n = 186, 89%).

**Table 1:** The demographics, characteristics, and clinical data.

	All		Non-sarcopenia		Sarcopenia		P-value
	n = 210		n = 186		n = 24		
Age, years	55	[45,66]	55	[45,65]	59	[46,67]	0.65
Sex							0.017*
Male	122	(58%)	114	(61%)	8	(33%)	
Female	88	(42%)	72	(39%)	16	(67%)	
Height, cm	164	[157,170]	165	[158,170]	157	[153,159]	<0.001*
Weight, kg	60	[51,69]	61	[54,71]	45	[42,49]	<0.001*
Body mass index, kg/m^2^	22	[20,25]	23	[20,25]	19	[17,21]	<0.001*
Dialysis vintage, months	19	[6,67]	17	[6,61]	45	[14,83]	0.025*
Donor type							0.051
Living-donor	174	(83%)	158	(85%)	16	(67%)	
Deceased-donor	36	(17%)	28	(15%)	8	(33%)	
ABO-incompatible kidney transplantation	47	(22%)	42	(23%)	5	(21%)	1.00
Calcineurin inhibitor							0.42
Tacrolimus	108	(51%)	98	(53%)	10	(42%)	
Cyclosporin	102	(49%)	88	(47%)	14	(58%)	
Antimetabolite or everolimus							0.25
Mycophenolate mofetil	150	(71%)	130	(70%)	20	(83%)	
Everolimus	45	(21%)	43	(23%)	2	(8.3%)	
Mizoribine	10	(4.8%)	8	(4.3%)	2	(8.3%)	
Azathioprine	5	(2.4%)	5	(2.7%)	0	(0.0%)	
Time after transplant, months	85	[43,135]	85	[43,133]	89	[51,170]	0.46
Hypertension	178	(85%)	161	(87%)	17	(71%)	0.086
Diabetes mellitus	47	(22%)	43	(25%)	4	(17%)	0.65
Hemoglobin, g/L	13	[12,14]	13	[12,14]	13	[12,14]	0.94
Fasting blood glucose, mg/dL	97	[89,110]	97	[89,110]	95	[88,102]	0.18
C-reactive protein, mg/dL	0.06	[0.02, 0.16]	0.06	[0.02, 0.17]	0.04	[0.01, 0.08]	0.083
Serum creatinine, mg/dL	1.3	[1.0, 1.6]	1.3	[1.0, 1.6]	1.1	[0.9, 1.3]	0.028*
Serum cystatin C, mg/dL	1.4	[1.1, 1.6]	1.4	[1.1, 1.6]	1.4	[1.1, 1.6]	0.68
Estimated glomerular filtration rate, ml/min/1.73 m^2^	54	[43,70]	54	[42,70]	55	[44,71]	0.67
HbA1c, %	5.8	[5.5, 6.3]	5.8	[5.5, 6.3]	5.7	[5.5, 6.1]	0.36
Total body water/lean mass, %	73.9	[73.6, 74.2]	73.9	[73.6, 74.2]	73.7	[73.6, 74.0]	0.063
Phase angle, °	4.8	[4.4, 5.3]	4.8	[4.4, 5.4]	4.3	[3.9, 4.6]	<0.001*
Handgrip strength, kg	28	[20,34]	29	[22,35]	17	[14,18]	<0.001*
Skeletal muscle mass index, kg/m^2^	7.1	[6.1, 8.1]	7.4	[6.5, 8.1]	5.4	[5.1, 5.6]	<0.001*
Gait speed, m/s	1.3	[1.2, 1.4]	1.4	[1.2, 1.5]	1.2	[1.1, 1.3]	0.002*

Categorical variables were expressed as count (percentage) and continuous variables were expressed as median [interquartile range]. Categorical variables were compared using chi-squared test, and continuous variables were compared using Mann-Whitney U-test. *p < 0.05.

**Figure 1:**
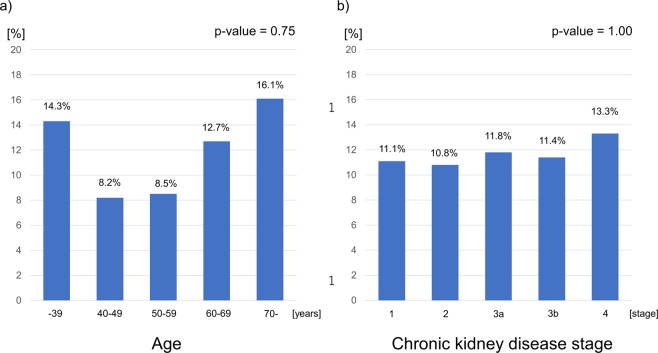
The prevalences of sarcopenia (**a**) in each age groups and (**b**) in each chronic kidney disease stage groups.

As shown in Fig. 1a, the prevalence of sarcopenia in −39, 40–49, 50–59, 60–69 and 70- age groups were 14.3% (4/28), 8.2% (4/49), 8.5% (4/47), 12.7% (7/55) and 16.1% (5/31), respectively. There was no relationship between age and sarcopenia (p = 0.75). The prevalences of sarcopenia in each CKD stage were the range of 10.8–13.3% regardless of CKD stage as shown in Fig. 1b. Similarly, there was no relationship between CKD stage and sarcopenia (p = 1.00).

### Comparisons between the sarcopenia and non-sarcopenia groups

As shown in Table 1, in the sarcopenia group, 16 (67%) were female, which was significantly more than that of the non-sarcopenia group (39%). The median dialysis vintage of the sarcopenia patients was 45 (IQR 14–83) months and longer than that of the non-sarcopenia patients (17 months). The median BMI of the sarcopenia patients was 19 (IQR 17–21) kg/m^2^, which was significantly lower than that of the non-sarcopenia patients (23 kg/m^2^). The median PhA of the sarcopenia patients was 4.3 (IQR 3.9–4.6°), which was lower than that of the non-sarcopenia patients (4.8°).

### Nutritional factors associated with sarcopenia

The results of multivariable logistic regression analysis evaluating nutritional factors associated with sarcopenia are shown in Table 2. BMI (for IQR difference of 5.2 units, odds ratio (OR) 0.14, 95% confidence interval (CI) 0.05–0.41, p < 0.001) and PhA (for IQR difference of 0.96 units, OR 0.36, 95%CI 0.16–0.82, p = 0.015) were significantly associated with sarcopenia after adjustment for potential confounders. The logistic regression models after penalized were internally validated and the estimated optimism was less than 0.2, indicating that there was no evidence of overfitting.

**Table 2:** Multivariable logistic regression analysis evaluating nutritional factors associated with sarcopenia.

Variables	Q1	Q3	OR (95%CI)	p-value
Age, years	45	66	0.82 (0.32–2.10)	0.68
Sex, female	—	—	1.35 (0.44–4.09)	0.60
Dialysis vintage, months	6	67	1.23 (0.84–1.78)	0.28
Time after transplant, months	44	135	1.20 (0.65–2.21)	0.56
Diabetes mellitus	—	—	0.92 (0.22–3.85)	0.91
Hemoglobin, g/dL	12	14	2.00 (0.93–4.33)	0.077
C-reactive protein, mg/dL	0.02	0.16	0.97 (0.82–1.15)	0.70
Estimated glomerular filtration rate, mL/min/1.73 m^2^	43	70	1.07 (0.49–2.36)	0.86
**Nutrition factors**				
Body mass index, kg/m^2^	20	25	0.14 (0.05–0.41)	<0.001*
Phase angle,°	4.4	5.3	0.36 (0.16–0.82)	0.015*

Q1, first quartile; Q3, third quartile; OR, odds ratio for the Q3 vs. Q1; CI, confidence interval. *p < 0.05.

**Figure 2:**
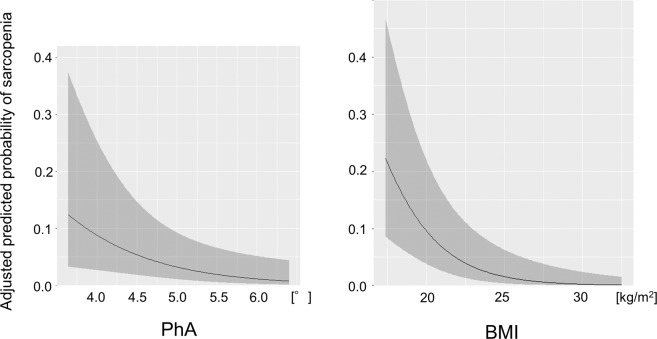
The predicted probability of sarcopenia based on PhA or BMI. The predicted probability and 95% confidence interval of sarcopenia based on PhA or BMI as nutritional factor after adjustment for age, sex, C-reactive protein, dialysis vintage, time after transplant, diabetes mellitus, hemoglobin, estimated glomerular filtration rate and the other nutritional factor are shown by the black line and the gray band, respectively. PhA, phase angle; BMI, body mass index.

Figure 2 shows the predicted probability of sarcopenia based on PhA or BMI after adjustment using multivariable logistic regression model. Both PhA and BMI were negatively correlated with sarcopenia.

### The discrimination performance of nutritional factors for sarcopenia

The area under the bootstrap receiver-operating characteristic curve (AUC-ROC) was 0.83 for BMI and 0.73 for PhA (Table 3). The optimal BMI and PhA cutoff value in order to detect sarcopenia was 20.5 kg/m^2^ and 4.46° according to the ROC using bootstrap method (Table 3). The area under the precision-recall curve (AUC-PR) was 0.97 for BMI and 0.96 for PhA (Table 3).

**Table 3:** Receiver operating characteristic curve and precision-recall curve for the nutritional factors to estimate the probability of sarcopenia.

Variables	Bootstrap ROC curve				PR curve
	AUC	Threshold	Sensitivity	Specificity	AUC
Body mass index	0.83	20.5	0.81	0.74	0.97
Phase angle	0.73	4.46	0.74	0.70	0.96

ROC, receiver operating characteristic; PR, precision-recall; AUC, area under the curve; threshold, the best cut-off score with Youden index.

## Discussion

In this single-center cross-sectional study, we demonstrated that the prevalence of sarcopenia based on the criteria of AWGS was 11.1% and PhA and BMI was negatively correlated with sarcopenia in kidney transplant recipients. PhA ≤ 4.46° and BMI ≤ 20.5 kg/m^2^ may therefore be used to increase the pretest probability of sarcopenia in kidney transplant recipients.

The present study showed that PhA was negatively correlated with sarcopenia in kidney transplant recipients. PhA is a parameter calculated from reactance and resistance, which are measured by BIA. Reactance expresses the capacity of cell membranes to store energy and is positively correlated with not only the quantity of cells but also the integrity of cell membranes in the body. Resistance expresses the volume of water compartments and is negatively correlated with the quantity of body fluids. Thus, PhA is regarded as a nutritional status indicator^15^. It is also an excellent predictor of morbidity and mortality in HIV, kidney disease, cancer, and geriatric patients^19–22^. Basile C *et al*. reported that PhA was positively correlated with muscle strength and mass in elderly patients^16^. Kilic MK *et al*. demonstrated that PhA was negatively correlated with sarcopenia and the optimal PhA cutoff value to detect sarcopenia was ≤4.55° in the elderly^17^. This result was almost the same as our results in kidney transplant recipients. However, Dos Reis AS *et al*. showed that PhA was associated with only handgrip strength (HGS), and not with other sarcopenia components and sarcopenia in kidney transplant recipients^23^. They divided kidney transplant recipients into two groups according to the first PhA tercile and investigated the association of PhA with sarcopenia and sarcopenia components. The difference between their PhA value (5.8° for women and 6.2° for men) and our optimal PhA cutoff value (4.46°) may explain the difference in the results between the two studies.

Our study showed that BMI was also negatively correlated with sarcopenia in kidney transplant recipients. It is well known that BMI is associated with sarcopenia^24–26^. The sarcopenia diagnostic criteria have components such as skeletal muscle mass index (SMI) and HGS adjusted for BMI^27^. Landi F *et al*. reported that BMI higher than 21 kg/m^2^ had significantly lower odds for sarcopenia than BMI lower than 21 kg/m^2^ (OR 0.76; 95% CI 0.64–0.90)^24^. Senior HE *et al*. demonstrated that BMI was negatively correlated with sarcopenia^25^. Kim H *et al*. showed that BMI lower than 21.0 kg/m^2^ was significantly associated with the development of sarcopenia in a longitudinal study^26^. Our study revealed that the optimal BMI cutoff value in order to detect sarcopenia was 20.5 kg/m^2^. Sarcopenia can coexist with obesity, and this state is referred to as sarcopenic obesity^28^. BMI combined with PhA may therefore be useful for the detection of sarcopenia in kidney transplantation.

Because the accuracy of the ROC may be uncertain due to the imbalanced data, we added the AUC-PR results. Although the criteria of threshold based on the PR curve is ambiguous even if the threshold based on the ROC was used, the precision was 0.9 or higher and recall was 0.7 or higher. Therefore, we believe that it can be well classified using the threshold of the ROC. Moreover, the AUC values for PhA and BMI had enough power to detect sarcopenia. These results suggested that BMI and PhA are beneficial factors in clinical practice to identify sarcopenia patients in kidney transplant recipients.

The percentage of kidney transplant recipients with sarcopenia was 11.1% in this study population, whose median age was 55 (IQR 46–66) years. Several reports have been made on the prevalence of sarcopenia in kidney transplant recipients, ranging from 11.8 to 49.6%^22,29,30^. Ozkayar N *et al*. reported that 34 out of 166 (20.5%) kidney transplant recipients, with a mean age of 37.9 ± 11.9 years, had sarcopenia based on only HGS^29^. Yanishi M *et al*. reported that 6 out of 51 (11.8%) kidney transplant recipients, with a mean age of 46.2 ± 12.8 years, had sarcopenia based on the AWGS criteria^30^. Dos Reis AS *et al*. reported that 64 out of 129 (49.6%) kidney transplant recipients, with a mean age was 47.8 ± 11.8 years, had sarcopenia based on the EWGSOP1 criteria^23^. This wide range of sarcopenia prevalence kidney transplant recipients may owe to the differences in sarcopenia definitions and population demographics such as age, sex, race, and comorbidities. In another kidney transplant population in which 50.3% had sarcopenia based on the EWGSOP1 criteria, 19%, 39%, 1.6%, and 5.5% were diagnosed as sarcopenia by the EWGSOP2 criteria defined as HGS + SMI, HGS + appendicular skeletal muscle mass (ASM), five times sit to stand (5STS) + SMI, and 5STS + ASM, respectively^31^. The prevalence of sarcopenia is known to increase with the degree of renal function impairment^32,33^. Moon SJ *et al*. reported that 5.6% of their study population, 11,625 subjects aged 40 years or older, had sarcopenia, and according to the stage of CKD, the prevalence of sarcopenia was 4.3%, 6.3%, and 15.4% in CKD 1, 2, and 3–5, respectively^33^. However, in our kidney transplant patients, the prevalence of sarcopenia was 11.1%, 10.4%, and 11.4% in CKD 1, 2, and 3–5, respectively, and renal graft function was not associated with sarcopenia.

In the present study, we evaluated renal graft function using the CKD-EPI 2012 equation combined with serum creatinine and serum cystatin C. Serum creatinine is affected by muscle mass or protein intake, while serum cystatin C is affected by inflammation or immunosuppression therapy. eGFR (estimated glomerular filtration rate) calculated using the CKD-EPI 2012 equation combined with creatinine and cystatin C may therefore be appropriate for the assessment of renal function in the elderly as well as in kidney transplant recipients^34–36^.

After adjustment for potential confounders, nutritional markers were associated with sarcopenia, while age and renal graft function were not in our kidney transplant patients. These results suggest that the impact of nutrition on sarcopenia is greater than that of aging or renal dysfunction in kidney transplant recipients. A low protein diet is recommended for kidney transplant recipients, because high protein intake may lead to damage to their renal grafts. However, an excessive low protein diet may lead to a decrease in the quality of life and to mortality in kidney transplant recipients, especially the elderly, so that it might be necessary to reconsider nutritional therapies for kidney transplant recipients.

Our study has several limitations. First, we could not reveal a causal relationship between sarcopenia and nutritional markers because of the cross-sectional design of our study. Second, this study was performed in a single center in Japan, and these results should not be generalized to subjects of other races or nationalities. Third, we evaluated muscle mass without CT and MRI, which are the gold standards.

In conclusion, our results showed that PhA and BMI may be beneficial factors in clinical practice to identify sarcopenia in kidney transplant recipients.

## Patients and Methods

### Study design and participants

A single-center cross-sectional study was conducted at Osaka City University Hospital between October 2018 and February 2019. The inclusion criteria were (1) clinical stability (defined as no hospitalization needed since the last check-up or within a month) and (2) more than a year post-transplant, while the exclusion criteria were (1) refusal to participate in this study, (2) missing data on diagnostic criteria for sarcopenia, and (3) not first kidney transplant (4) abnormal hydration status, which is <69 or >75% of total body water/lean mass. This study was approved by the Ethics Committee of Osaka City University Graduate School of Medicine (No. 3859). All patients provided written informed consent for participation in the study, and all the procedures were in accordance with the Helsinki Declaration of 2000.

### Sarcopenia diagnosis

Sarcopenia was diagnosed by low muscle mass and either low muscle strength or low physical performance according to the AWGS criteria^5^. Low muscle strength was defined as HGS <26 kg for males and <18 kg for females. Low muscle mass was defined as SMI <7.0 kg/m^2^ for males and <5.7 kg/m^2^ for females. Low functional capacity was defined as gait speed <0.8 m/s.

### Muscle strength and physical performance measurements

Gait speed was measured by the time to walk 10 m, and the average time of two trials was taken. HGS was measured on both hands alternately twice, with a Smedley hand-held dynamometer at a standing position with elbow in full extension, and the best of the two attempts were recorded. All gait speed and HGS were measured by the same single observer.

### Bioimpedance measurements

The ASM, total body water, lean mass, and PhA were measured by BIA using the InBody S10 (InBody Co, Ltd., Seoul, Korea), which has a tetrapolar eight-point tactile electrode system and six different frequencies (1 kHz, 5 kHz, 50 kHz, 250 kHz, 500 kHz, 1000 kHz). Measurements were taken while the patients laid flat on a bed in the supine position with their limbs away from the body midline. Reactance and resistance were measured at 50 kHz. PhA was calculated according to the following formula: PhA [°] = arctangent (reactance/resistance)*(180/π)^37^. SMI was calculated as ASM in kilograms divided by height in meters squared.

### Biochemical measurements

Blood samples were drawn in the morning after overnight fasting. Hemoglobin, C-reactive protein, fasting blood glucose, hemoglobin, HbA1c, serum creatinine, serum cystatin C were measured using the JCA-BM6070 (JEOL Ltd., Tokyo, Japan) automatic biochemical analyzer.

### Data collection

The demographics (age, sex), characteristics (dialysis vintage, time after transplant, transplantation type, presence of diabetes mellitus) and clinical data (hemoglobin, C-reactive protein, fasting blood glucose, hemoglobin, HbA1c, serum creatinine, serum cystatin C) were collected from electronic medical records. Body weight and height were measured wearing light clothing and without shoes, and BMI was calculated as weight in kilograms divided by height in meters squared. eGFR was calculated using the CKD-EPI 2012 equation combined with serum creatinine and serum cystatin C^34^.

### Statistical analysis

Categorical variables were expressed as count and percentage, and continuous variables were expressed as median and IQR. Categorical variables were compared using chi-squared test, and continuous variables were compared using Mann-Whitney U-test.

For primary analysis, to assess the relationship between sarcopenia and PhA or BMI as nutritional factors, the multivariable logistic regression model was used with adjustment for age, sex, C-reactive protein, dialysis vintage, time after transplant, diabetes mellitus, hemoglobin, eGFR and the other nutritional factor (for sarcopenia, non-sarcopenia = 0 and sarcopenia = 1). To avoid overfitting, the regression model was limited to two nutritional factors and 8 covariates, and penalized maximum likelihood estimation was performed to allow shrinkage for effect of each variable. The selection of covariates was made a priori according to expert opinion and previous literature, namely age^37,38^, sex^38^, C-reactive protein^39,40^, diabetes mellitus^41,42^, dialysis vintage^42^, time after transplant, hemoglobin^43^, and eGFR^31^. Long-term use of immunosuppressive agents including steroids and calcineurin inhibitors has a negative effect on the muscles of kidney transplant recipients^44,45^. Optimism assesses the magnitude of overfitting of logistic regression model (a value less than 0.2 is considered as good), and was calculated using C-statistics by 150 times of bootstrap sampling.

For secondary analysis, the discrimination performance for sarcopenia was assessed by AUC-ROC and AUC-PR. We reported bootstrap bias-corrected AUC as the validated measure of the predictive performance of each nutritional factor. The sensitivity and specificity were calculated by using the best cut-off score for the nutritional factors with the Youden index for the ROC. The statistical analyses were performed using R version 3.5.1 (R Foundation for Statistical Computing, Vienna, Austria). A two-sided p < 0.05 was considered statistically significant.

## Data Availability

The datasets generated during and/or analysed during the current study are available in the figshare repository 10.6084/m9.figshare.7992851.
